# Endoscopic Ultrasound and Gastric Sub-Epithelial Lesions: Ultrasonographic Features, Tissue Acquisition Strategies, and Therapeutic Management

**DOI:** 10.3390/medicina60101695

**Published:** 2024-10-15

**Authors:** Marzia Varanese, Marco Spadaccini, Antonio Facciorusso, Gianluca Franchellucci, Matteo Colombo, Marta Andreozzi, Daryl Ramai, Davide Massimi, Roberto De Sire, Ludovico Alfarone, Antonio Capogreco, Roberta Maselli, Cesare Hassan, Alessandro Fugazza, Alessandro Repici, Silvia Carrara

**Affiliations:** 1 Department of Surgery, Sapienza University of Rome, 00185 Rome, Italy; marzia.varanese@gmail.com; 2 Division of Gastroenterology and Digestive Endoscopy, Humanitas Research Hospital—IRCCS, Rozzano, 20089 Milano, Italy; 3 Department of Biomedical Sciences, Humanitas University, Pieve Emanuele, 20072 Milano, Italy; 4 Gastroenterology Unit, Department of Medical Sciences, University of Foggia, 71100 Foggia, Italy; 5 Gastroenterology and Hepatology, The University of Utah School of Medicine, Salt Lake City, UT 84113, USA

**Keywords:** gastric subepithelial lesions, endoscopic ultrasound, fine needle biopsy, gastrointestinal stromal tumors, Fine needle aspiration, artificial intelligence

## Abstract

*Background and objectives*: Subepithelial lesions (SELs) of the gastrointestinal (GI) tract present a diagnostic challenge due to their heterogeneous nature and varied clinical manifestations. Usually, SELs are small and asymptomatic; generally discovered during routine endoscopy or radiological examinations. Currently, endoscopic ultrasound (EUS) is the best tool to characterize gastric SELs. *Materials and methods*: For this review, the research and the study selection were conducted using the PubMed database. Articles in English language were reviewed from August 2019 to July 2024. *Results*: This review aims to summarize the international literature to examine and illustrate the progress in the last five years of endosonographic diagnostics and treatment of gastric SELs. *Conclusions*: Endoscopic ultrasound is the preferred option for the diagnosis of sub-epithelial lesions. In most of the cases, EUS-guided tissue sampling is mandatory; however, ancillary techniques (elastography, CEH-EUS, AI) may help in both diagnosis and prognostic assessment.

## 1. Introduction

Subepithelial lesions (SELs) of the gastrointestinal (GI) tract present a diagnostic challenge due to their heterogeneous nature and varied clinical manifestations.

As their name says, they originate beneath the mucosal layer, either from the muscularis mucosa, submucosa, or muscularis propria. They are generally discovered during routine endoscopy or radiological examinations with an incidence of approximately 0.36% [1].

Usually, SELs are small and asymptomatic; however, in some cases, they can cause dysphagia, evident or occult gastrointestinal bleeding with chronic anemia, and compression [2]. Prognosis varies from benign and indolent to malignant and potentially aggressive neoplasia, such as gastrointestinal stromal tumors (GIST) and neuroendocrine tumors (NET) [3]. The majority of the SELs are benign, but 15% are malignant [4].

Endoscopically, they appear as intraluminal protuberances covered with normal or ulcerated overlying mucosa. Chromoendoscopy and narrow-band imaging are not useful since they don’t originate from the mucosa, which is usually normal [5]. Conventional endoscopy cannot identify the etiology of the lesions since the biopsies taken with forceps are often insufficient and too superficial to identify their histopathologic characteristics.

Currently, endoscopic ultrasound (EUS) is the best tool to characterize gastric SELs. EUS is used to determine the layer of origin, the size, echogenicity, vascularization, and connection with surrounding structures [6]. Fine needle aspiration (FNA) or fine needle biopsy (FNB) guided by EUS allows us to obtain a cytological and histological diagnosis of subepithelial gastric lesions.

This study aims to review the international literature to examine and illustrate the progress in the last five years of endosonographic diagnostics and the treatment of gastric SELs.

## 2. Methods

For this review, the research and the study selection were conducted using the PubMed database. Articles in English language were reviewed from August 2019 to July 2024. We made the research using the following terms: “gastric subepithelial lesions”, “gastric submucosal lesions”, “gastrointestinal stromal tumors”, “endosonography”, “fine needle biopsy”, “fine needle aspiration”, “contrast enhanced EUS”, “EUS elastography”, “artificial intelligence EUS”, and “MIAB”.

## 3. Gastric Subepithelial Lesions

Gastric subepithelial lesions are classified into non-neoplastic lesions, including inflammatory fibroid lesions, varices, lipomas, duplication cysts, and ectopic pancreas and neoplastic lesions, such as gastrointestinal stromal tumors (GIST), leiomyomas, lymphomas, schwannomas, glomus tumors, neuroendocrine tumors, and lymphangiomas [3] (Table 1).

A mesenchymal tumor is the one with the highest incidence of gastric localization (approx. 54%) [4]. GISTs are the most common mesenchymal tumors of the GI tract [7]. They are mainly identified in the stomach as intramural nodules covered by normal mucosa, sometimes appearing as umbilicated lesions with central ulceration. At EUS, they are hypoechoic, round-shaped lesions arising from the muscularis propria [8]. These are mostly benign tumors where the size of the lesion and the mitotic count are prognostic factors for malignancy potential [9]. For this reason, it is important to perform tissue sampling using EUS-guided biopsy. According to the National Comprehensive Cancer Network (NCCN), the treatment of GISTs ≥ 2 cm and symptomatic GISTS ≤ 2 cm is surgical resection, while small and asymptomatic lesions should be followed up [10].

Leiomyomas are another type of mesenchymal tumors, most frequent in the esophagus, arising from the muscularis propria. Most frequently they are asymptomatic, but bulky lesions can rarely cause dysphagia. These lesions appear endosonographically small (<5 cm), homogenous, and hypoechoic with regular borders [11]. Considering that leiomyomas, unlike GISTs, are benign lesions, it is important to perform the differential diagnosis between these two types of tumors.

Another category of submucosal gastric lesions is neuroendocrine tumors, which account for 0.5% of all tumors, of which the majority are found in the gastrointestinal tract [12]. Carcinoid tumors, the most common type of NET, originate from enterocromaffin cells and are slow-growing, mainly located in the stomach. Gastric carcinoids are usually asymptomatic and may be incidentally discovered at GI endoscopy. NETs may originate from the muscularis mucosa and need a forceps biopsy for the diagnosis. In case of lesions originating from the submucosal layer, EUS is required to evaluate the depth of invasion and the presence of lymph nodes [13]. The NCCN recommends surveillance for tumors ≤ 20 mm in size and surgical resection for larger ones [10]. The American Society for Gastrointestinal Endoscopy (ASGE) recommends endoscopic resection of types 1, 2, and 3 gastric carcinoids ≤ 1 cm and surgical resection of type 3 gastric carcinoids ≥ 1 cm and all type 4 carcinoids [14].

A rare neoplastic lesion in the stomach is schwannoma, a benign nerve sheath tumor found in 0.2% of all gastric neoplasms [11,15]. They usually involve the submucosa and muscularis propria and are discovered incidentally. On EUS, they appear as small, hypoeocogenic, homogeneous lesions with distinct and homogeneous margins [8].

The ultrasound appearance of benign gastric subepithelial lesions is considered diagnostic, so tissue sampling is usually not necessary. The most frequent benign lesions are lipomas that may appear throughout the gastrointestinal tract as solitary, yellow-colored neoformations. In the EUS, lipomas present as hyperechogenic, homogeneous masses arising from the submucosal layer with low growth [16].

Duplication cysts are congenital anomalies whose gastric localization is rare, accounting for only 2–8% of all duplication cysts located in the gastrointestinal tract [17]. It is often an occasional finding, and endosonographically they present as anecogenic round lesions with defined margins located in the third layer of the gastric wall.

EUS has also found a role in the evaluation of esophageal and gastric varices. Endosonographically, they present as round or tortuous anechogenic structures at the level of the submucosal layer. EUS in combination with the color-Doppler technique is a non-invasive method that identifies and differentiates varices from gastric folds [11,18].

The last benign, sub-epithelial lesion is the ectopic or aberrant pancreas, a pancreatic tissue that can be found elsewhere without any connection to the normal pancreas. They are most frequently diagnosed in the stomach asymptomatically and can rarely cause pancreatitis and cancer. On endoscopic evaluation, they present as hypoechogenic or mixed structures with anechogenic areas inside, corresponding to ductal structures. Differential diagnosis with carcinoid tumors may be difficult due to their similar endosonographic appearance [19,20].

## 4. Endoscopic Ultrasound (EUS)

Endoscopic ultrasound represents a significant step forward in diagnoses and characterization of gastric SELs. Due to its high resolution, EUS is recommended by ESGE guidelines as the best tool to characterize SEL features (size, location, originating layer, echogenicity, shape, and vascularization) [5]. EUS also makes it possible to differentiate between external compressions and subepithelial lesions [21,22], with a sensitivity of 92% in recognizing extrinsic compressions [23], but EUS alone is not able to distinguish among all types of SELs.

Some SELs, such as lipomas, varices, or ectopic pancreas, can be established by EUS features alone with high accuracy. In cases of a non-diagnostic endosonographic feature, given the malignant potential of some of the SELs, histological examination is mandatory [5,24]. Biopsies performed with conventional forceps are usually not diagnostic, because the overlying mucosa is normal. EUS tissue acquisition techniques mainly include fine needle aspiration (FNA) and fine needle biopsy (FNB) (ESGE guidelines). EUS-guided tissue sampling is indicated for subepithelial lesions > 20 mm by EUS-FNA, EUS-FNB, or mucosal incision-assisted biopsy (MIAB) and/or with high-risk features on EUS (heterogeneity, echogenic foci, or irregular margins) [5,6]. Studies show that the results of FNB-EUS and MIAB are comparable for lesions > 20 mm and have higher diagnostic results than EUS-FNA [25]. For subepithelial lesions < 20 mm, some recent studies suggest that EUS-FNB and MIAB have the highest diagnostic yield compared to EUS-FNA, with an advantage for MIAB in lesions < 20 mm [26,27].

### 4.1. Contrast-Enhanced Harmonic EUS (CEH-EUS)

In the recent years, contrast-enhanced harmonic endoscopic ultrasound, by using a contrast agent the enhances the microperfusion, has been used for the characterization of solid tumors, including SELs. This new technique detects echo signals from microbubbles in vessels with very slow flow without artifacts.

CEH-EUS has a central role in the differential diagnosis between GISTs and leiomyomas, allowing the estimation of the malignancy potential of gastrointestinal stromal tumors [28].

**Figure 1:**
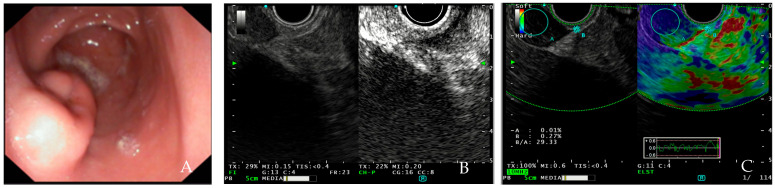
(**A**) EUS showing GIST in the antrum of stomach; (**B**) GIST in contrast-enhanced endoscopic ultrasound showing hypervascularization and displaying inhomogeneous contrast-uptake; (**C**) elastography of GIST located in the duodenum showing blue pattern. [source Division of Gastroenterology and Digestive Endoscopy, Humanitas Research Hospital—IRCCS, Rozzano, Milan].

Factors associated with a high malignant risk are abnormal intratumoral blood vessels, heterogeneous perfusion pattern, and the presence of non-enhancing spots. The washout of the contrast agent is predominantly slow in the GISTs and NETs and fast in the majority of the lipomas and leiomyomas. The lesion can be evaluated according to the level of enhancement. There are three patterns used to quantify within-lesion blood flow on CEH-EUS: hypo-enhancement, iso-enhancement, and hyper-enhancement. The generated image shows a hyper-enhancement pattern in gastrointestinal stromal tumors with a sensitivity of 78–100%, a specificity of 60–100% an accuracy of 60–100% and a hypo-enhancement pattern in benign subepithelial tumors [29,30] (Figure 1).

The washout of the contrast agent is predominantly slow in the GISTs and NETs and fast in the majority of the lipomas and leiomyomas. In some cases, it may be difficult to differentiate GISTs from leiomyomas based on subjective evaluation of contrast patterns alone. Artificial intelligence could increase diagnostic accuracy in discriminating between GISTs and leiomyomas.

CEH-EUS is, therefore, a minimally invasive imaging modality that can be used as an additional diagnostic tool and may also be adopted in assessing the response of treatment by examining blood flow in GISTs, but further studies are needed. High expectations are also relied on CH-EUS for the monitoring of antiangiogenic treatments of GISTs and the evaluation of gastrointestinal neuroendocrine tumors (NETs).

### 4.2. EUS-Elastography

Real-time EUS elastography (EUS-E) is an advanced imaging technique that can be conducted using a standard EUS probe connected to a processor equipped with a dedicated software that measures tissue stiffness and adds more diagnostic value to EUS. This evaluation is performed by overlaying the image color according to the tissue stiffness into B mode. Blue color represents hard lesions, green color for the intermediate tissue, and red color for soft tissue [31]. Besides the qualitative evaluation of the color scale, it is possible to perform a semi-quantitative assessment, the strain ratio (SR), which is calculated from the ratio between the stiffness of a region of interest and the stiffness of the adjacent area The higher the SR value, the higher will be the tissue stiffness. Hue histogram is another good parameter for the semi-quantitative evaluation of solid lesions; it graphically displays the range of colors (hues) in the elastography image, indicating tissue elasticity from softest to hardest along the x-axis and the count of pixels at each level of elasticity on the y-axis.

A higher in-tissue stiffness can be associated with many diseases, including cancer or potentially malignant lesions. Changes in tissue stiffness can be associated with various pathologies, including cancer [32,33].

Although both contrast-enhanced EUS and elastography may be useful in clinical practice, only few data support their use in the diagnostic management of SELs. For these reasons, ESGE guidelines suggest that CH-EUS can be used for characterization of SELs in the upper digestive tract and estimation of the malignant potential of GISTs (Figure 1), but it cannot replace EUS tissue acquisition. Moreover, ESGE suggests that there is insufficient evidence to recommend EUS-E in the diagnosis and management of SELs [5].

### 4.3. Artificial Intelligence in Endoscopic Ultrasound

For several years, artificial intelligence has enabled numerous advances in the diagnosis and treatment of gastrointestinal tract diseases.

AI should increase the ability to identify potentially progressive lesions at an early stage, thus reducing their evolution into cancer. Some artificial intelligence tools not only identify lesions but also predict their histology, suggesting to the endoscopist whether they are adenomas (potentially dangerous anomalies) or non-adenomatous lesions.

Furthermore, an AI system can help reduce diagnostic and therapeutic errors that are inevitable in human clinical practice.

AI was initially used to improve early-stage diagnosis of colorectal cancer during colonoscopy but later found a role in upper GI diseases. In recent years, artificial intelligence (AI) has been gradually recognized as a diagnostic method for gastric subepithelial lesions in endosonography. In 2020, Minoda et al. studied the diagnostic accuracy of EUS-AI-based on gastric SELs for GISTs and non-GISTs, indicating that EUS-AI has an accurate diagnosis for GISTs ≥ 20 mm [34]. It has also been observed that lesion size increases the diagnostic accuracy of EUS-AI [35].

In a recent meta-analysis, the ability to predict the malignant potential of GISTs by EUS-AI was evaluated by classifying low, intermediate, and high risk GISTs, and it was shown that AI has a high accuracy in predicting malignant potential [36].

Hirai et al., in a multicenter retrospective study, developed an EUS-AI model for the most frequent SELs, including GIST, leiomyoma, NET, schwannoma, and ectopic pancreas, and evaluated the diagnostic accuracy of the model and endoscopists. The EUS-AI was shown to have a diagnostic accuracy of 86.1% for SELs, higher than the endoscopist’s experience; furthermore, the EUS-AI was reported to have a high sensitivity and accuracy in distinguishing GISTs from non-GISTs with 98.8% and 89.3%, respectively, showing higher rates compared to endoscopists [37].

The use of EUS-AI has opened up new opportunities in the diagnosis, treatment, and follow-up of SELs by increasing the sensitivity, specificity, and accuracy of endosonographers, differentiating these lesions from others. Further studies are required for its validation.

## 5. Fine Needle Biopsy (FNB)

**Figure 2:**
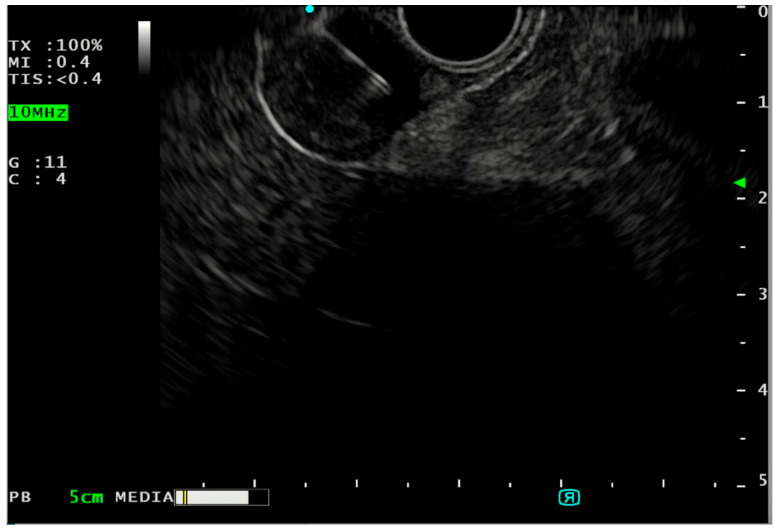
FNB of GIST. [source Division of Gastroenterology and Digestive Endoscopy, Humanitas Research Hospital—IRCCS, Rozzano, Milan].

FNB is widely used for tissue acquisition in SELs (Figure 2). The tissue core samples obtained with the 3rd generation FNB needles (fork-tip, Franseen tip needles) are more appropriate for histological evaluation, molecular diagnostics, and immunohistochemical stain than cytological samples [38]. The European Society of Gastrointestinal Endoscopy (ESGE) guidelines consider EUS-FNB as first choice along with MIAB for the diagnosis of SELs [5], in contrast to the American College of Gastroenterology (ACG), which recommends EUS-FNB or EUS-FNA with rapid on-site cytological evaluation (ROSE) as the first diagnostic step when EUS-FNB is not available [2]. Studies have shown that EUS-FNB has high diagnostic accuracy in identifying GISTs (89–93.8%) compared to EUS-FNA (37–75%) [39,40].

The tissue core samples obtained with the 3rd generation FNB needles (fork-tip, Franseen tip needles) are more appropriate for histological evaluation, molecular diagnostics, and immunohistochemical stain than cytological samples [38]. The European Society of Gastrointestinal Endoscopy (ESGE) guidelines consider EUS-FNB as first choice along with MIAB for the diagnosis of SELs [5], in contrast to the American College of Gastroenterology (ACG), which recommends EUS-FNB or EUS-FNA with rapid on-site cytological evaluation (ROSE) as the first diagnostic step when EUS-FNB is not available [2]. Studies have shown that EUS-FNB has high diagnostic accuracy in identifying GISTs (89–93.8%), compared to EUS-FNA (37–75%) [39,40].

EUS-FNA with ROSE allows immediate assessment of the acceptability of the sample obtained, thus reducing the number of needle passes to be performed [41]. However, it requires the presence of a cytopathologist during the procedure, who may not be present in all facilities and significantly increases the cost of these procedures. A recent review demonstrated that EUS-FNB has a higher diagnostic yield for SELs than EUS-FNA with ROSE, thus reducing the need for multiple attempts to obtain tissue [42].

In a recent paper, it has been reported how, through FNB needles, it is possible to measure high-frequency impedance (H-impedance), which allows us to differentiate between GIST and non-GIST, especially in lesions < 20 mm [43].

Needle size (22G vs. 19G reverse bevel tip needles) seems to have no impact on FNB sensitivity, while the FNB sensitivity (using the 22G Franseen tip needle) is significantly higher when visible white tissue cores of >4 mm in length can be identified in the specimen on on-site assessment [5].

A randomized study has compared the technique of macroscopic on-site evaluation (MOSE) during EUS-guided fine-needle biopsy and EUS-FNB performed with three needle passes [44]. MOSE was performed by the endoscopist by evaluating the collected material considering a white/yellowish aggregate core longer than 10 mm adequate. No significant differences were found between EUS-FNB with MOSE and conventional EUS-FNB in terms of diagnostic accuracy, sample appropriateness, and rate of adverse events. MOSE adequately evaluates the sample by reducing the number of needle passes.

## 6. Fine Needle Aspiration (FNA)

Although EUS-FNB is superior to EUS-FNA in the current American guidelines, this is still a valid option, especially when combined with ROSE [2]. The diagnostic accuracy of EUS-FNA for the detection of gastric SELs ranges from 60 to 90% [45,46]. Although it is a frequently used technique in gastric SELs, the amount of cytological material taken is often insufficient for immunohistochemical staining to differentiate lesions [47,48]. A recent meta-analysis compared EUS-FNA with ROSE versus the other techniques, showing that EUS-FNA is a valid technique when rapid on-site cytological evaluation is available [27]. The diagnostic accuracy of EUS-FNA depends on several factors but mainly on lesion size. Sekine et al., comparing FNA and FNB needles, found no difference in accuracy in lesions > 20 mm (FNA vs. FNB, 75% vs. 77.8%), but, in lesions < 20 mm, the accuracy of the FNB needle was significantly higher (FNA vs. FNB, 72.7% vs. 100%) [49]. Although it is considered a safe procedure, EUS-FNA, like the FNB, may have complications, especially bleeding. Such complication depends on the needle that is used in the procedure or on the failure in suspending antithrombotic drugs [50]. It is also important to perform the procedure safely, paying attention to the large blood vessels adjacent to the lesion.

## 7. Mucosal Incision-Assisted Biopsy (MIAB)

An alternative for tissue sampling is MIAB. It is considered a valid method when a diagnosis cannot be made with EUS-FNA/FNB [2]. MIAB involves tissue sampling by performing an ‘open’ biopsy during a gastroscopy and does not require an experienced endoscopist in EUS. MIAB requires longer procedure time than guided EUS techniques in gastric lesions and is also associated with a higher risk of bleeding [51]. Recent studies have compared MIAB to EUS-FNB showing how the diameter of the SELs can influence the diagnostic yield [27,42]. In subepithelial gastric lesions < 20 mm, MIAB is more successful than the other methods. In a recent meta-analysis, MIAB ranked as the best intervention for lesions < 20 mm (SUCRA score 0.86 for adequacy and 0.91 for accuracy), and EUS-FNB was only slightly superior to EUS-FNA [27]. It has been also evaluated how submucosal tunneling during an MIAB procedure may preclude endoscopic resection, so a possible strategy for lesions < 20 mm could be to proceed directly with endoscopic removal.

## 8. Other Diagnosis Methods

Currently, endoscopic ultrasound is the most sensitive imaging modality for the evaluation of gastric SELs, but there are also other diagnostic procedures that can evaluate them. Computed tomography (CT) is a useful imaging technique in the evaluation of abdominal lesions and is less invasive but cannot determine the layer of origin of subepithelial lesions. Kim et al. found that the accuracy of EUS in the diagnosis of gastric SELs was 64.2%, while CT had an accuracy of 50.9% [52]. Certain CT features allow precise differentiation between GISTs and non-GISTs [53]. Magnetic resonance imaging (MRI) and positron emission tomography (PET) with 18F fluorodeoxyglucose can be useful in differentiating GISTs from non-GISTs and high-risk GISTs from low-risk GISTs [54]. PET has a sensitivity of 80% and a specificity of 66.7% in differentiating low-risk GISTs from high-risk GISTs but cannot distinguish leiomyomas and schwannomas due to their high signal [54]. According to current guidelines, PET is not recommended in differential diagnosis but has an important role in treatment choice and planning [13].

## 9. Management and Treatment

The management of benign subepithelial lesions such as leiomyomas, schwannoma, lipomas, and ectopic pancreas do not require surveillance, unlike GISTs for which a different management is required due to their malignant potential. Guidelines recommend resection for GISTs with high-risk EUS features or with a size > 2 cm [2,5]. In contrast, in GISTs < 2 cm in size, surveillance is recommended given the low risk of malignancy [2,5]. Surveillance with EUS for lesions between 1 and 2 cm is recommended at 1–2 year intervals, while for lesions smaller than 1 cm, a 2–3-year interval is recommended [5,37]. However, the European Society of Medical Oncology (ESMO) suggests resection regardless of lesion size [34].

The endoscopic techniques used for resection of gastric lesions are endoscopic submucosal resection (ESMR), endoscopic submucosal dissection (ESD), endoscopic full-thickness resection (EFTR), retractligate-unroof biopsy (RLUB), and endoscopic submucosal tunnelling resection (STER). The choice of endoscopic resection depends on various factors, including lesion characteristics, site, and evidence of deeper tissue involvement.

ESMR is a technique adopted for lesions with submucosal invasion [55].

ESD is performed for larger lesions and is a minimally invasive technique most frequently used in GISTs and NETs within specific size limits and without suspected locoregional involvement [56]. ESD may be difficult for lesions originating from the muscle layer, and, for lesions > 5 cm in size, increasing the risk of perforation by up to 20% can be considered a valid alternative to surgery [57]. The feasibility of ESD for the treatment of GISTs must be evaluated mainly based on the location of the lesion. ESD seems to be a good option for lesions that protrude into the gastric lumen with close contact with the muscularis tunic, while GISTs that are located in the center of the gastric wall or that have extraluminal growth are candidates for surgery or EFTR [56].

Recently, a new technique called endoscopic full-thickness resection has been developed for resection of deep submucosal lesions that cannot undergo ESMR and ESD. This technique requires the use of a device that includes a plastic cap preloaded with an over-the-scope clip (OTSC), an integrated snare, and a grasper. During the procedure, the lesion is marked, and the tissue is brought into the plastic cap with the grasper. The OTSC is applied, and the snare resects the lesion above the applied OTSC. It is considered a safe and effective procedure that allows intraluminal resection with reduced bleeding risks [58]. Further studies comparing these new endoscopic resection techniques are needed, since they may change the management of SELs in the future.

STER is a new, minimally invasive endoscopic therapeutic technique for submucosal tumors of the upper gastrointestinal tract that can provide a definitive histological diagnosis. This method creates a submucosal tunnel while maintaining the mucosa intact, thus reducing the risk of abdominal infections and leakage in the post-operative period. The procedure can only be performed for lesions < 35 mm that do not originate from a deep layer of the muscularis propria, because this would increase the risk of complications [59].

## 10. Conclusions

Endoscopic ultrasound is the preferred option for the diagnosis of sub-epithelial lesions. In most of the cases, EUS-guided tissue sampling is mandatory; however, ancillary techniques (elastography, CEH-EUS) may help in both diagnosis and prognostic assessment. Due to the variety of their biological behavior, the management of these lesions varies from oncological therapies to follow-up. A dedicated multidisciplinary team should be always involved in case of potentially malignant lesions when considering the different therapeutic options. As is the case for gastric adenocarcinoma, also for this type of lesion, it is essential to define centers of excellence, well-organized multidisciplinary networks, and centralization of high-risk procedures. Furthermore, advanced training for new generations should be the priority [60].

## Figures and Tables

**Table 1:** EUS description of SELs.

Subepithelial Lesion	Layer of Origin	Echogenicity	Location in GI Tract	Malignant Potential
Lymphoma (5)	2nd/3rd/4th	Hypoechoic	Anywhere in the GI tract	Yes
Lymphangioma (5)	3rd	Anechoic, no Doppler signal, with internal septa	Small intestine	No
Schwannoma (8)	4th	Hypoechoic, homogenous, sometimes with marginal halo	Stomach (body)	No
GIST (9)	2nd/4th	Hypoechoic, hypervascular, heterogeneous with cystic space or echogenic foci	Stomach	Yes
Leiomyoma (11)	2nd/4th	Hypoechoic, rarely multifocal fine margin	Esophagus	No
Varices (11)	3rd	Anechoic with Doppler signal	Esophagus	No
Neuroendocrine tumor (12)	1st/2nd/3rd	Hypoechoic/hyperechoic	Stomach, duodenum, rectum	Yes
Lipoma (16)	3rd	Hyperechoic, homogenous	Anywhere in the GI tract	No
Duplication cyst (17)	3rd/external	Anechoic, no Doppler signal	Esophagus	Very Rarely
Heterotopic pancreas (19)	3rd/4th	Hypoechoic, heterogenous, with cysts or ducts inside	Stomach (antrum)	Very rarely
Glomus tumor (38)	3rd/4th	Hypo-hyperechoic, hypervascular with internal echo	Anywhere in the GI tract	Yes

## Data Availability

Not applicable.
